# Maternal vomiting during early pregnancy and cardiovascular risk factors at school age: the Generation R Study

**DOI:** 10.1017/S2040174419000114

**Published:** 2019-09-02

**Authors:** Sunayna Poeran - Bahadoer, Vincent W. V. Jaddoe, Olta Gishti, Iris J. Grooten, Oscar H. Franco, Albert Hofman, Eric A. P. Steegers, Romy Gaillard

**Affiliations:** 1 The Generation R Study Group, Erasmus MC, University Medical Center Rotterdam, Rotterdam, the Netherlands; 2 Department of Epidemiology, Erasmus MC, University Medical Center Rotterdam, Rotterdam, the Netherlands; 3 Department of Pediatrics, Erasmus MC, University Medical Center Rotterdam, Rotterdam, the Netherlands; 4 Department of Obstetrics and Gynecology, Academical Medical Center Amsterdam, Amsterdam, the Netherlands; 5 Department of Obstetrics and Gynecology, Erasmus MC, University Medical Center Rotterdam, Rotterdam, the Netherlands

**Keywords:** Childhood body mass index, hyperemesis gravidarum, lipids, obesity, vomiting

## Abstract

**Background:**

Evidence suggests that low birth weight and fetal exposure to extreme maternal undernutrition is associated with cardiovascular disease in adulthood. Hyperemesis gravidarum, a clinical entity characterized by severe nausea and excess vomiting leading to a suboptimal maternal nutritional status during early pregnancy, is associated with an increased risk of adverse pregnancy outcomes. Several studies also showed that different measures related to hyperemesis gravidarum, such as maternal daily vomiting or severe weight loss, are associated with increased risks of adverse fetal pregnancy outcomes. Not much is known about long-term offspring consequences of maternal hyperemesis gravidarum and related measures during pregnancy. We examined the associations of maternal daily vomiting during early pregnancy, as a measure related to hyperemesis gravidarum, with childhood cardiovascular risk factors.

**Methods:**

In a population-based prospective cohort study from early pregnancy onwards among 4,769 mothers and their children in Rotterdam, the Netherlands, we measured childhood body mass index, total fat mass percentage, android/gynoid fat mass ratio, preperitoneal fat mass area, blood pressure, lipids, and insulin levels. We used multiple regression analyses to assess the associations of maternal vomiting during early pregnancy with childhood cardiovascular outcomes.

**Results:**

Compared with the children of mothers without daily vomiting during early pregnancy, the children of mothers with daily vomiting during early pregnancy had a higher childhood total body fat mass (difference 0.12 standard deviation score [SDS]; 95% confidence interval [CI] 0.03–0.20), android/gynoid fat mass ratio (difference 0.13 SDS; 95% CI 0.04–0.23), and preperitoneal fat mass area (difference 0.10 SDS; 95% CI 0–0.20). These associations were not explained by birth characteristics but partly explained by higher infant growth. Maternal daily vomiting during early pregnancy was not associated with childhood blood pressure, lipids, and insulin levels.

**Conclusions:**

Maternal daily vomiting during early pregnancy is associated with higher childhood total body fat mass and abdominal fat mass levels, but not with other cardiovascular risk factors. Further studies are needed to replicate these findings, to explore the underlying mechanisms and to assess the long-term consequences.

## Introduction

Maternal undernutrition during pregnancy may lead to increased risks of cardiovascular disease in the offspring in later life.^1^ This hypothesis is mainly based on studies linking low birth weight and fetal exposure to extreme maternal undernutrition to the development of diseases in adulthood.^2–4^ Whether suboptimal maternal nutritional status during pregnancy in contemporary populations also directly affects the development of cardiovascular risk factors in the offspring remains unclear.

Hyperemesis gravidarum, a clinical entity affecting approximately 0.3–3.6% of all pregnancies, is characterized by severe nausea and excess vomiting leading to a suboptimal maternal nutritional status during early pregnancy and is associated with an increased risk of adverse pregnancy outcomes.^5–15^ Hyperemesis gravidarum represents the extreme of the spectrum of maternal excess vomiting and extreme nausea during early pregnancy. Several studies also showed that different measures related to hyperemesis gravidarum, such as maternal daily vomiting or severe maternal weight loss, are associated with increased risks of adverse pregnancy outcomes, including preterm birth and small size for gestational age at birth.^16–18^ A previous study among 3,165 Dutch mothers and their children suggested that maternal hyperemesis gravidarum, which was measured as severe weight loss during early pregnancy, was associated with a higher blood pressure in childhood.^18^ No differences were present in childhood body mass index, glucose, triglycerides, total cholesterol, high-density lipoprotein (HDL), and low-density lipoprotein (LDL)-cholesterol. In this study, no information about maternal vomiting during early pregnancy was available.

Based on these previous findings, we hypothesized that the children of mothers with daily vomiting during early pregnancy may also have a higher risk of adverse cardiovascular outcomes in childhood. Daily maternal vomiting may lead to suboptimal maternal nutritional status, which may subsequently lead to suboptimal fetal nutrient supply and developmental adaptations in response to a suboptimal fetal environment.^2,3^ These fetal developmental adaptations may permanently affect fetal growth, fetal adipocyte development and fat deposition and structure, and the function of cardiovascular organs. This may subsequently lead to an increased risk of adverse birth outcomes and obesity and adverse cardiovascular outcomes in childhood and adulthood.^19,20^ Therefore, we examined, in a population-based prospective cohort study among 4,769 mothers and their children, the associations of maternal vomiting during early pregnancy with childhood body mass index, total body fat mass, android/gynoid fat mass ratio, abdominal preperitoneal fat mass area, blood pressure, lipids and insulin levels, measured at 6 years. We also explored whether these associations were explained by maternal, birth, or childhood characteristics.

## Methods

### Study design

**Fig. 1.**
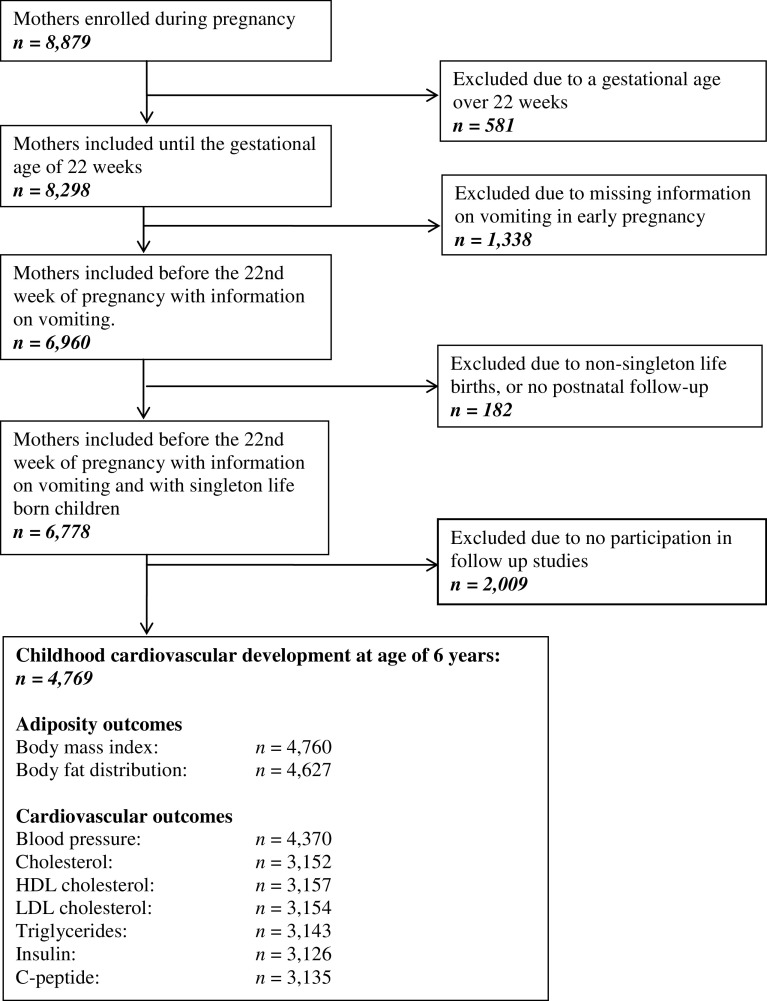
Flow chart of the study population.

This study was embedded in the Generation R Study, a population-based prospective cohort study from early fetal life onwards in Rotterdam, the Netherlands.^21,22^ The study was approved by the local Medical Ethical Committee (MEC 198.782/2001/31). Pregnant women were enrolled between 2001 and 2005. Written informed consent was obtained from all participating mothers. In total, 8,879 mothers were enrolled during pregnancy. From our analyses, we excluded those who enrolled after 22 weeks of gestation (*n* = 581) and those who did not have information on vomiting during early pregnancy available (*n* = 1,338). Of the included women, 6,778 delivered singleton live born children, of which 4,769 mothers and their children had childhood follow-up measurements available (Fig. 1).

### Maternal vomiting and nausea during early pregnancy

Information on maternal vomiting and nausea during early pregnancy was obtained via questionnaires at enrollment in the study.^21^ Maternal vomiting and nausea was reported in five categories based on the number of times that pregnant women vomited or had nausea during early pregnancy: daily; a few days a week; once a week; less than once a week; never. We compared maternal daily vomiting during early pregnancy, an indicator of hyperemesis gravidarum, with other maternal vomiting categories. This approach is in line with previous studies.^6–13,23^ Primary analyses were performed using maternal vomiting, and sensitivity analyses were performed using maternal nausea, across similar categories, instead of maternal vomiting to examine whether the associations differed between maternal vomiting and maternal nausea.

### Childhood adiposity and cardiovascular outcomes

All children were invited for detailed body fat and cardiovascular follow-up measurements at the age of 6. We measured height and weight without shoes and heavy clothing at a dedicated research facility. Height was measured to the nearest millimeter with a stadiometer (Holtain Limited, Crosswell, Crymych, UK). Weight was measured to the nearest gram using an electronic scale (SECA 888, Almere, The Netherlands), and body mass index (kg/m^2^) was calculated.^24^

Total body fat mass was measured with a dual-energy X-ray absorptiometry (DXA) scanner (iDXA, General Electrics – Lunar, 2008, Madison, WI, USA) and analyzed with the Encore software v.12.6.^25^ DXA can accurately detect whole-body fat mass within <0.25% coefficient of variation. Total body fat mass (kg) was calculated as a percentage of total body weight (kg) measured by DXA. Android/gynoid fat mass ratio was also calculated, which reflects the waist-to-hip ratio.^26^

Preperitoneal fat mass was used as a proxy for visceral fat and was measured using abdominal ultrasound examinations with ultrasound LOGIQ E9 (GE Medical System, Wauwatosa, WI, USA) and ATL-Philips Model HDI 5000 (Seattle, WA, USA), as described in detail previously.^27–29^ Briefly, a linear (L12-5 MHz) transducer was placed perpendicular to the skin surface on the median upper abdomen. We scanned longitudinally from the xiphoid process to the navel along the midline (linea alba). Preperitoneal fat mass areas were measured as areas of 2 cm length along the midline starting from the reference point in direction of the navel.^27^

We measured systolic and diastolic blood pressure at the right brachial artery, four times with 1-min intervals, using a validated automatic sphygmanometer (Datascope Accutor Plus™; Paramus, NJ, USA).^30^ A cuff was selected with a cuff width approximately 40% of the arm circumference and long enough to cover 90% of the arm circumference. We calculated mean systolic and diastolic blood pressure values using the last three blood pressure measurements.

We obtained 30-min fasting venous blood samples and measured total cholesterol, low-density lipoprotein cholesterol, high-density lipoprotein cholesterol, triglycerides, insulin, and C-peptide levels, using Cobas 8000 analyzer (Roche, Almere, the Netherlands).

### Covariates

Maternal age and prepregnancy weight were obtained at enrolment. Maternal height (cm) was measured and prepregnancy body mass index (kg/m^2^) was calculated. We defined gestational weight gain as the difference between weight before pregnancy and weight measured at 30 weeks of gestation (median 30.2; 95% range 28.5–32.6).^21^ Information on maternal educational level, ethnicity, parity, smoking, and folic acid supplement use was obtained via questionnaires. First-trimester nutritional intake was recorded via a food frequency questionnaire.^31^ Information on pregnancy complications, mode of delivery, gestational age, length and weight at birth, and sex was available from medical records.^32,33^ We constructed gestational age-adjusted standard deviation scores (SDS) for birth weight using North European growth standards.^34^ These gestational age-adjusted SDS for birth characteristics represent the equivalent of *z*-scores. Infant growth was measured in community health centers according to standardized procedures at 24 months (median 24.8 months; 95% range 23.4–28.1).^21^ We created age- and sex-adjusted SDS of these infant anthropometrics within our study population using Dutch reference growth charts.^35^ We obtained information about breastfeeding, TV watching, and timing of introduction of solid foods via questionnaires.^21^

### Statistical analysis

First, we examined differences in subject characteristics between mothers with and without daily vomiting during early pregnancy with ANOVA and chi-squared tests. Second, to provide further insight into the correlation of measures related to hyperemesis gravidarum with maternal weight, nutritional status, and birth outcomes, we assessed the associations between maternal daily vomiting and nausea during early pregnancy with multiple parameters of maternal nutritional status and birth outcomes using univariate linear regression models. Third, we examined the associations of maternal daily vomiting during early pregnancy with childhood outcomes and the role of potential mediators using linear regression models. For these analyses, we used different linear regression models: (1) A basic model including child’s age and sex. (2) A confounder model, which additionally included covariates selected on their associations with the outcomes of interest or a change in effect estimate >10%. Covariates included were maternal age, educational level, ethnicity, prepregnancy weight, parity, smoking, folic acid supplement use, diet, delivery mode, and pregnancy complications. We included childhood height as a covariate in all models focusing on fat mass outcomes. (3) Intermediate models, which additionally included potential intermediates (gestational weight gain; birth characteristics including gestational age and weight at birth; infant growth from birth until 2 years of age; and infant lifestyle including breastfeeding duration, age at introduction of solid foods, and TV watching). (4) A fully adjusted model including all confounders and all potential intermediate covariates. Supplementary Figure S1 depicts an overall conceptual model with all potential confounders and intermediate covariates for clarification on the strategy of analysis and for interpretation of the results. For all analyses, non-normally distributed childhood outcome variables were log-transformed. We constructed SDS values ([observed value – mean]/SD) for childhood outcomes to enable a comparison of effect estimates. Since no significant interactions with fetal sex and birth weight were present after taking into account multiple testing, no further stratified analyses were performed. We performed sensitivity analyses by comparing maternal daily vomiting with never vomiting; by assessing a dose–response relationship using maternal vomiting categories as a continuous variable, from category “never” up to category “daily”; and by performing similar analyses using daily nausea during early pregnancy. In order to reduce potential bias associated with missing data and to maintain statistical power, we performed multiple imputations of missing covariates by generating five independent datasets using the Markov Chain Monte Carlo method after which the pooled effect estimates were calculated. All analyses were performed using SPSS version 21.0 for Windows (SPSS Inc, Chicago, IL, USA).

## Results

### Subject characteristics

The characteristics of included participants are shown in Table 1. In total, 5.2% of all women vomited daily during early pregnancy. Compared with mothers without daily vomiting during early pregnancy, mothers with daily vomiting during early pregnancy were younger, more frequently less educated, and more frequently of non-European origin (*p* < 0.05). Results of the non-response analysis are shown in Supplemental Table S1. Compared with mothers included in the analyses, those lost to follow-up were more likely to be older, less educated, and more often vomited daily during early pregnancy; their children had a lower gestational age at birth, lower birth weight, and were less frequently breastfed.

**Table 1.** Maternal and childhood characteristics (*N* = 4,769)^1^

	No maternal daily vomiting during early pregnancy (*n* = 4,306)	Maternal daily vomiting during early pregnancy (*n* = 463)	*P*-value
**Maternal characteristics**			
Age, years	30.6 (4.9)	28.1 (5.0)	<0.05
Height, cm	168.3 (7.2)	165.0 (7.3)	<0.05
Weight, kg	66.3 (12.3)	67.5 (14.4)	0.06
Body mass index, kg/m^2^	23.4 (4.1)	24.9 (5.0)	<0.05
Total gestational weight gain, kg	15.4 (5.5)	12.6 (6.7)	<0.05
Gestational age at intake, weeks	14.3 (2.9)	14.6 (2.9)	<0.05
Parity (nulliparous), %	60.1	52.7	<0.05
Education (higher education), %	50.3	20.9	<0.05
Ethnicity (European), %	67.1	33.9	<0.05
Smoking during pregnancy (yes), %	25.3	24.8	0.26
Folic acid supplement use (yes), %	79.6	57.0	<0.05
Total energy intake, kcal	2,069 (545)	1,945 (617)	<0.05
Daily nausea during early pregnancy, %	26.7	94.1	<0.05
Pregnancy complications			
Gestational hypertensive disorders, %	6.1	6.5	0.74
Gestational diabetes, %	0.9	1.6	0.15
Birth and infant characteristics			
Gestational age at birth, weeks	39.9 (1.7)	39.8 (1.7)	<0.05
Birth weight, g	3443 (547)	3364 (532)	<0.05
Gestational age-adjusted birth weight (SDS)	–0.06 (0.99)	–0.19 (0.98)	<0.05
Male sex, %	49.7	51.6	0.43
Cesarean delivery, %	12.6	10.2	0.14
Breastfeeding duration, months	4.6 (3.9)	3.9 (3.7)	<0.05
Introduction of solid foods (before 6 months), %	89.4	88.7	0.17
Television watching (>2 hours/day), %	17.3	33.9	<0.05
Child characteristics			
Age at follow-up, years	6.2 (0.5)	6.3 (0.7)	<0.05
Infant weight growth from birth until 2 years, g	9,581 (1440)	9,838 (1712)	<0.05
Body mass index, kg/m^2^	16.2 (1.8)	16.8 (2.4)	<0.05
Total body fat mass, %	0.25 (0.06)	0.27 (0.06)	<0.05
Android/gynoid fat mass ratio, %	0.25 (0.06)	0.27 (0.08)	<0.05
Abdominal preperitoneal fat mass area, cm^2^	0.45 (0.44,0.46)	0.54 (0.51,0.58)	<0.05
Systolic blood pressure, mmHg	102.4 (8.1)	103.8 (8.8)	<0.05
Diastolic blood pressure, mmHg	60.5 (6.7)	61.4 (7.3)	<0.05
Cholesterol, mmol/L	4.2 (0.6)	4.2 (0.6)	0.69
HDL-cholesterol, mmol/L	1.3 (0.3)	1.4 (0.3)	0.13
LDL-cholesterol, mmol/L	2.4 (0.6)	2.4 (0.5)	0.61
Triglycerides, mmol/L	1.1 (1.0,1.1)	1.1 (1.0,1.1)	0.71
Insulin, pmol/L	137.9 (134.2,141.5)	146.5 (134.1,158.9)	0.16
C-peptide, nmol/L	1.0 (1.0,1.1)	1.0 (1.0,1.1)	0.93

1 Values represent mean (standard deviation), median (95% range), or percentages. Differences in subject characteristics between the groups were evaluated using one-way ANOVA for continuous variables and chi-squared tests for proportions.

Univariate associations of maternal daily vomiting and nausea during early pregnancy with maternal weight status were studied using multiple parameters of maternal nutritional status and birth outcomes shown in Supplementary Tables S2, S3, and S4. Maternal daily vomiting and nausea during early pregnancy was associated with a higher maternal prepregnancy weight and BMI, but with lower total gestational weight gain. No significant differences in trimester-specific weight gain were present. Among the women with daily vomiting and nausea during early pregnancy, total caloric, fat, and protein intakes tended to be lower, compared with women without daily vomiting and nausea during early pregnancy. The children of mothers with daily vomiting during early pregnancy had a lower birth weight and a lower gestational age at birth, compared with the children of mothers without daily vomiting during early pregnancy. These effects were weaker for maternal daily nausea during early pregnancy.

### Maternal vomiting and childhood adiposity outcomes

Table 2 shows the associations of maternal daily vomiting during early pregnancy with childhood body fat distribution at the age of 6. In the confounder model, compared with the children of mothers without daily vomiting during early pregnancy, the children of mothers with daily vomiting during early pregnancy had a higher childhood total body fat mass (difference 0.12 SDS; 95% confidence interval [CI] 0.03–0.20), android/gynoid fat mass ratio (difference 0.13 SDS; 95% CI 0.04–0.23), and abdominal preperitoneal fat mass area (difference 0.10 SDS; 95% CI 0–0.20), but no significant difference in childhood body mass index was present (difference 0.07 SDS; 95% CI –0.02 to 0.16). Additional adjustment for birth characteristics did not explain the observed associations, but adjustment for infant growth slightly attenuated the associations. In the fully adjusted model, maternal daily vomiting during early pregnancy was associated with a higher childhood body mass index, total body fat mass, android/gynoid fat mass ratio, and preperitoneal fat mass (*p* < 0.05). The effect estimates were not affected when we restricted the analyses to daily vomiting versus never vomiting only. Supplementary Table S5 shows that in the dose–response analysis, only maternal daily vomiting during early pregnancy was associated with higher childhood body fat mass measures. Also, similar tendencies were observed when using daily nausea during early pregnancy instead of daily vomiting (results not shown).

**Table 2.** Maternal daily vomiting and childhood general and abdominal fat outcomes (*N* = 4,760)^1^

	Childhood fat outcomes			
	Body mass index (*n* = 4,760)	Total body fat mass (*n* = 4,627)	Android/gynoid fat mass ratio (*n* = 4,627)	Abdominal preperitoneal fat mass area (*n* = 3,838)
Basic model^2^	0.25 (0.16, 0.35)*	0.33 (0.24, 0.41)*	0.26 (0.16, 0.35)*	0.24 (0.14, 0.34)*
Confounder model^3^	0.07 (–0.02, 0.16)	0.12 (0.03, 0.20)*	0.13 (0.04, 0.23)*	0.10 (0, 0.20)*
Mediator models^4^				
Gestational weight gain	0.10 (0, 0.19)*	0.12 (0.04, 0.21)*	0.14 (0.04, 0.23)*	0.11 (0.01, 0.22)*
Birth characteristics	0.08 (–0.01, 0.17)	0.12 (0.03, 0.20)*	0.13 (0.03, 0.23)*	0.10 (0, 0.21)*
Infant growth	0.06 (–0.03, 0.14)	0.10 (0.02, 0.18)*	0.12 (0.02, 0.21)*	0.09 (–0.02, 0.19)
Infant lifestyle	0.07 (–0.02, 0.16)	0.12 (0.03, 0.20)*	0.13 (0.03, 0.23)*	0.10 (0, 0.20)*
Fully adjusted model^5^	0.08 (0, 0.17)*	0.12 (0.03, 0.20)*	0.11 (0.02, 0.21)*	0.10 (0, 0.20)*

^1^Values are regression coefficients (95% confidence intervals) that reflect the difference for each body fat measure per standard deviation score change between the children of mothers with and without daily vomiting during early pregnancy. Estimates are based on multiple imputed data. ^2^Basic model is adjusted for child’s sex and age at outcome measurements. ^3^Confounder models include maternal age, educational level, ethnicity, prepregnancy weight, parity, smoking, folic acid supplement use, diet, delivery mode, and pregnancy complications. Models for fat mass are additionally adjusted for childhood height. ^4^Intermediate models are additionally adjusted for each potential intermediate (1. gestational weight gain; 2. birth characteristics: gestational age at birth and birth weight; 3. infant growth: growth from birth until 2 years of age; 4. infant lifestyle: breastfeeding duration, age at introduction of solid foods, and TV watching). ^5^Fully adjusted models include all potential confounders and intermediates. *P < 0.05.

### Maternal vomiting and childhood cardiovascular risk factors

Table 3 shows that in the basic model, the children of mothers with daily vomiting during early pregnancy had a higher systolic blood pressure, but not diastolic blood pressure, compared with the children of mothers without daily vomiting during early pregnancy (difference in systolic blood pressure: 0.11 SDS; 95% CI 0.01–0.20). Additional adjustment for confounding factors attenuated the associations into non-significant. No differences were observed in childhood total cholesterol, triglycerides, and insulin levels between the children of mothers with or without daily vomiting during early pregnancy. Supplementary Table S6 shows that in the dose–response analysis, no associations of maternal daily vomiting during early pregnancy with childhood cardiovascular measures were present.

**Table 3.** Maternal daily vomiting during early pregnancy and childhood cardiovascular risk factors (*N* = 4,370)^1^

	Childhood cardiovascular risk factors				
	Systolic blood pressure (*n* = 4,370)	Diastolic blood pressure (*n* = 4,370)	Total cholesterol (*n* = 3,152)	Triglycerides (*n* = 3,143)	Insulin (*n* = 3,126)
Basic model^2^	0.11 (0.01, 0.20)*	0.09 (–0.01, 0.19)	0 (–0.12, 0.12)	0.03 (–0.09, 0.15)	0.07 (–0.05, 0.19)
Confounder model^3^	0.03 (–0.08, 0.12)	0.02 (–0.09, 0.12)	–0.04 (–0.16, 0.09)	0.03 (–0.09, 0.16)	0.07 (–0.05, 0.20)
Mediator models^4^					
Gestational weight gain	0.03 (–0.08, 0.13)	0.03 (–0.07, 0.14)	–0.04 (–0.16, 0.09)	0.02 (–0.11, 0.15)	0.09 (–0.04, 0.22)
Birth characteristics	0.02 (–0.08, 0.12)	0.01 (–0.09, 0.11)	–0.04 (–0.16, 0.08)	0.03 (–0.10, 0.15)	0.07 (–0.05, 0.20)
Infant growth	0.02 (–0.08, 0.12)	0.01 (–0.09, 0.11)	–0.04 (–0.16, 0.09)	0.03 (–0.09, 0.16)	0.07 (–0.06, 0.19)
Infant lifestyle	0.03 (–0.08, 0.13)	0.01 (–0.09, 0.11)	–0.04 (–0.16, 0.08)	0.03 (–0.09, 0.16)	0.08 (–0.05, 0.20)
Fully adjusted model^5^	0.02 (–0.08, 0.12)	0.03 (–0.08, 0.13)	–0.04 (–0.17, 0.09)	0.02 (–0.11, 0.15)	0.09 (–0.04, 0.21)

^1^Values are regression coefficients (95% confidence intervals) that reflect the differences in childhood cardiovascular risk factors per standard deviation score change between the children of mothers with and without daily vomiting during early pregnancy. Estimates are based on multiple imputed data. ^2^Basic model is adjusted for child’s sex and age at outcome measurements. ^3^Confounder models include maternal age, educational level, ethnicity, prepregnancy weight, parity, smoking, folic acid supplement use, diet, delivery mode, and pregnancy complications. Models for fat mass are additionally adjusted for childhood height. ^4^Intermediate models are additionally adjusted for each potential intermediate (1. gestational weight gain; 2. birth characteristics: gestational age at birth and birth weight; 3. infant growth: growth from birth until 2 years of age; 4. infant lifestyle: breastfeeding duration, age at introduction of solid foods, and TV watching). ^5^Fully adjusted models include all potential confounders and intermediates. *P < 0.05.

## Discussion

In this prospective cohort study, we observed that daily maternal vomiting during early pregnancy was associated with a higher childhood total body fat mass, android/gynoid fat mass ratio, and abdominal preperitoneal fat mass. These associations were not explained by birth characteristics, but partly explained by higher infant growth rates. We did not observe associations of maternal daily vomiting during early pregnancy with childhood blood pressure, lipids levels, or insulin levels.

### Methodological considerations

We used a population-based prospective cohort design involving a large number of subjects who we studied from early fetal life onwards. Information on vomiting during early pregnancy was available for 6,960 mothers, of which 4,769 mothers and their children participated in childhood follow-up measurements. Mothers without offspring follow-up data available were more often less educated and from non-European descent, and more often vomited daily during early pregnancy. The observed associations would be biased if the associations of maternal vomiting during early pregnancy with childhood outcomes differed between those included and those exempted in the analyses. This seems unlikely, but cannot be excluded. However, our study group might indicate a selection toward a relatively healthy population, which could affect the generalizability of our results. We used daily maternal vomiting as an indicator of hyperemesis gravidarum. This approach is in line with previous observational studies.^6–13,23^ This indicator is one component related to hyperemesis gravidarum, but does not fully reflect a severe clinical diagnosis of hyperemesis gravidarum, which includes dehydration, hospitalization, electrolyte imbalance, nutritional deficiencies, and maternal weight loss. We observed that mothers with daily vomiting during early pregnancy had a lower total gestational weight gain and a lower total caloric intake. Due to the small number of mothers who vomited daily or had nausea and reported severe weight loss, we were not able to assess the associations of these combined maternal characteristics with childhood outcomes in our study. However, additional adjustment for gestational weight gain did not explain the observed associations of maternal daily vomiting or nausea with childhood outcomes. Further studies are needed to assess the associations of a clinical diagnosis of hyperemesis gravidarum as well as other maternal characteristics relating to hyperemesis gravidarum with long-term offspring consequences. Detailed information about several maternal and childhood sociodemographic and lifestyle-related factors was available in this study. Extensive adjustment for these sociodemographic and lifestyle-related determinants in our analyses did not explain the associations of maternal daily vomiting during early pregnancy with childhood body fat outcomes. However, because of the observational design, residual confounding due to other sociodemographic and lifestyle-related determinants, such as residential area, maternal physical activity or sedentary lifestyle and childhood nutritional intake, might still be an issue.

### Interpretation of main findings

An accumulating body of evidence suggests that suboptimal maternal nutrition during early pregnancy leads to an increased risk of cardiovascular disease in later life of the offspring.^1^ Low birth weight or fetal exposure to extreme maternal undernutrition is associated with cardiovascular and metabolic diseases in adulthood.^2,3^ Less is known about the long-term effects of milder and more common suboptimal maternal nutritional status during pregnancy.

Hyperemesis gravidarum, characterized by severe nausea and excess vomiting during early pregnancy, resulting in electrolyte, fluid, and nutrition imbalances and deficiencies, is a marker of suboptimal maternal nutritional status during early pregnancy.^36–38^ Previous studies have shown that hyperemesis gravidarum might lead to an increased risk of adverse outcomes during pregnancy.^5–15^ A study among 71,468 Norwegian mothers showed that children from hyperemetic mothers – defined as enduring nausea and vomiting starting before the 25th gestational week leading to hospitalization – had a lower gestational age at birth, but not a lower birth weight.^23^ Another study among 156,091 mothers in Canada showed that hyperemesis gravidarum – defined as severe nausea and vomiting occurring before the 24th gestational week leading to hospitalization – was associated with an increased risk of preterm birth, low birth weight, and small size for gestational age.^10^ Thus, hyperemesis gravidarum may adversely affect fetal development, leading to both preterm delivery and low birth weight.

Hyperemesis gravidarum represents the extreme of the spectrum of maternal excess vomiting and extreme nausea during pregnancy. Several studies have shown that other less extreme measures relating to hyperemesis gravidarum, such as maternal daily vomiting or severe weight loss, are also associated with an increased risk of adverse fetal pregnancy outcomes, including preterm delivery and small size at birth.^16–18^ Not much is known about the long-term consequences of maternal hyperemesis gravidarum and its related measurements with childhood adiposity and cardiovascular outcomes. We observed that maternal daily vomiting during early pregnancy was not associated with a higher body mass index of the offspring. Our results are in line with a previous cohort study among 3,165 Dutch mothers and their children.^18^ This study reported that the body mass index at 5 and 6 years of age was similar between the children of mothers with and without hyperemesis gravidarum, which was measured by a severe weight loss during early pregnancy.

During childhood, body mass index may not be an accurate marker of fat mass, as it provides no information on body fat distribution and cannot distinguish lean mass from fat mass. Previously we showed that detailed general and abdominal fat mass measures are, independent of body mass index, associated with cardiovascular risk factors at school age.^39^ Similarly, among adults, waist circumference, as a proxy for abdominal fat mass, is related to cardiovascular disease and mortality in adulthood, independent of body mass index.^40–42^ In the current study, we observed that maternal daily vomiting during early pregnancy was associated with a higher childhood total body fat mass, android/gynoid fat mass ratio, and abdominal preperitoneal fat mass. To the best of our knowledge, no other studies have examined these associations yet. Surprisingly, none of these associations were explained by gestational age or size at birth, even though both characteristics are known to be related to both general and abdominal fat mass in later life.^2,43^ Our findings were partly explained by infant growth, a well-known risk factor for adverse body fat distribution.^43,44^ Thus, our findings suggest that the children of mothers with daily vomiting during early pregnancy have higher total and abdominal fat mass levels, which may partly be explained by suboptimal nutrition during fetal life and compensatory growth acceleration during the postnatal period.

We did not observe the associations of maternal daily vomiting during early pregnancy with childhood blood pressure and blood lipids and insulin levels. In contrast, a previous study among 3,165 Dutch mothers and their children suggested that maternal hyperemesis gravidarum, defined as a severe maternal weight loss during early pregnancy, was associated with a higher blood pressure in childhood, but not with childhood total cholesterol and triglycerides.^18^ A study among 78 participants found that insulin sensitivity was 20% lower in the children of mothers with severe hyperemesis gravidarum, defined as intractable vomiting and electrolyte imbalances during early pregnancy leading to hospitalization.^5^ Differences in results might be due to the use of different measurements relating to hyperemesis gravidarum, differences in study populations, and adjustments for confounders. Altogether, the results of the present and previous studies do not suggest consistent associations of maternal vomiting during early pregnancy with offspring cholesterol, triglycerides, and insulin levels.

### Underlying mechanisms

The underlying pathophysiology of hyperemesis gravidarum is not known yet. From an evolutionary adaptation perspective, mild vomiting during pregnancy may be considered as a defensive strategy to expel harmful foods or spoiled or teratogenic substances.^6,45–47^ However, daily maternal vomiting may also lead to suboptimal maternal nutrition, which may subsequently lead to suboptimal fetal nutrition and developmental adaptations.^2^ These fetal developmental adaptations may permanently affect fetal growth, fetal adipocyte development and function, and the function of cardiovascular organs, predisposing to adverse body composition and an increased risk of obesity in later life. Importantly, none of the observed associations of maternal daily vomiting during early pregnancy with childhood total body and abdominal fat mass levels were explained by birth characteristics. This finding is in line with previous studies that focused on extreme maternal undernutrition, which showed that exposure to extreme maternal undernutrition during early gestation has an increased risk of obesity and cardiovascular disease in later life, independent of size at birth.^19,20,48^ Together, these findings might imply that the effects of maternal daily vomiting on offspring’s body composition may act through other mechanisms than size at birth, such as alterations in the levels of fetal micronutrients leading to epigenetic changes or endocrine alterations in the offspring.^18^ Further research is needed to obtain more insights into the causality and underlying mechanisms of the observed associations.

The effect estimates of the associations of maternal daily vomiting during early pregnancy with childhood total body and abdominal fat mass levels were small and are mainly of interest from an etiological perspective. However, since these may be important from a cardiovascular developmental perspective, their effects on the risk of cardiovascular disease should be further studied. Previous studies have shown that childhood body composition measures tend to track from childhood into adulthood.^49–52^ A study among 7,723 and 7,252 children aged 7 and 11 years, respectively, showed moderate tracking of fat mass throughout childhood.^50^ A study among 2,204 participants showed a strong 27-year tracking between childhood and adulthood measurements of body mass index.^49^ A study among 4,857 children and adolescents, aged 5–20 years, with a median age of 11 years, showed that childhood obesity was associated with increased rates of premature death from endogenous causes.^51^ Similarly, a study among 276,835 Danish schoolchildren showed that a higher childhood BMI across the full range was associated with an increased risk of coronary heart disease in adulthood.^52^ Thus, these studies suggest that even subclinical differences in childhood fat mass levels are related to the development of cardiovascular disease in later life. Further studies are needed to identify the long-term consequences of maternal hyperemesis gravidarum on offspring’s body fat and cardiovascular outcomes.

## Conclusions

Our results suggest that maternal daily vomiting during early pregnancy is associated with higher childhood total and abdominal fat mass levels, independent of size or gestational age at birth. Further studies are needed to replicate these findings, to explore the underlying mechanisms, and to assess the long-term consequences.

## Supplementary material

For supplementary material accompanying this paper visit https://doi.org/10.1017/S2040174419000114. click here to view supplementary material
