# Supplementary material for: Maternal vomiting during early pregnancy and cardiovascular risk factors at school age: the Generation R Study
Source: J Dev Orig Health Dis. 2019 Sep 2;11(2):118–26. doi: 10.1017/S2040174419000114 (PMC7282857; doi:10.1017/S2040174419000114)
Supplement: Supplementary file 1 [file S2040174419000114sup001.docx]

Supplementary Material

Maternal vomiting during early pregnancy and cardiovascular risk factors at school-age. The Generation R Study

Sunayna Poeran – Bahadoer MSc^1,2^ ([s.bahadoer@erasmusmc.nl](mailto:s.bahadoer@erasmusmc.nl)), Vincent W.V. Jaddoe MD, PhD^1,2,3^ ([v.jaddoe@erasmusmc.nl](mailto:v.jaddoe@erasmusmc.nl)), Olta Gishti MD, PhD^1,2^ ([o.gishti@erasmusmc.nl](mailto:o.gishti@erasmusmc.nl)), Iris J. Grooten MD, PhD^4^ ([i.j.grooten@amc.uva.nl](mailto:i.j.grooten@amc.uva.nl)), Oscar H. Franco MD, PhD^2^ ([o.franco@erasmusmc.nl](mailto:o.franco@erasmusmc.nl)), Albert Hofman MD, PhD^1,2^ ([a.hofman@erasmusmc.nl](mailto:a.hofman@erasmusmc.nl)), Eric A.P. Steegers MD, PhD^5^ ([e.a.p.steegers@erasmusmc.nl](mailto:e.a.p.steegers@erasmusmc.nl)), Romy Gaillard MD, PhD^1,2^ ([r.gaillard@erasmusmc.nl](mailto:r.gaillard@erasmusmc.nl))

Author affiliations:

1. The Generation R Study Group, Erasmus MC, University Medical Center Rotterdam, the Netherlands,
2. Department of Epidemiology, Erasmus MC, University Medical Center Rotterdam, the Netherlands,
3. Department of Pediatrics, Erasmus MC, University Medical Center Rotterdam, the Netherlands,
4. Department of Obstetrics and Gynaecology, Academical Medical Center Amsterdam, the Netherlands
5. Department of Obstetrics and Gynaecology, Erasmus MC, University Medical Center Rotterdam, the Netherlands

**Supplementary Table S1. Non-response analysis (N = 6,778)^1^**

|  | **No participation**  n = 2,009 | **Participation**  n = 4,769 | **P-value** |
| --- | --- | --- | --- |
| **Maternal Characteristics** |  |  |  |
| Age, years | 28.4 (5.4) | 30.4 (5.0) | P<0.05 |
| Height, cm | 166.8 (7.3) | 168.0 (7.3) | P<0.05 |
| Weight, kg | 65.7 (13.6) | 66.4 (12.5) | P<0.05 |
| Body mass index, kg/m^2^ | 23.6 (4.7) | 23.5 (4.2) | P=0.54 |
| Total gestational weight gain, kg | 14.8 (6.0) | 15.2 (5.6) | P=0.12 |
| Gestational age at intake, weeks | 14.7 (3.1) | 14.4 (2.9) | P<0.05 |
| Parity (Nulliparous), % | 54.7 | 59.4 | P<0.05 |
| Education (higher education), % | 34.2 | 47.5 | P<0.05 |
| Ethnicity (European), % | 50.1 | 63.9 | P<0.05 |
| Smoking during pregnancy (yes), % | 29.3 | 25.3 | P<0.05 |
| Folic acid supplement use (yes),% | 63.6 | 77.5 | P<0.05 |
| Total energy intake, kcal | 2,026 (588) | 2,059 (553) | P=0.06 |
| Daily nausea during early pregnancy, % | 36.1 | 33.3 | P=0.17 |
| Daily vomiting during early pregnancy, % | 13.5 | 9.7 | P<0.05 |
| **Pregnancy complications** |  |  |  |
| Gestational hypertensive disorders, % | 5.8 | 6.2 | P=0.57 |
| Gestational diabetes, % | 1.0 | 0.9 | P=0.79 |
| **Birth and infant characteristics** |  |  |  |
| Gestational age at birth, weeks | 39.7 (2.1) | 39.9 (0.7) | P<0.05 |
| Birth weight, g | 3385 (580) | 3436 (546) | P<0.05 |
| Male sex, % | 52.5 | 49.9 | P<0.05 |
| Caesarean delivery, % | 12.9 | 12.4 | P=0.57 |
| Breastfeeding duration, months | 4.2 (3.7) | 5.0 (3.8) | P<0.05 |
| Introduction of solid foods (before 6 months), % | 89.7 | 89.3 | P=0.84 |
| Television watching (more than 2 hours/day), % | 19.1 | 18.7 | P=0.84 |

^1^Values represent means (standard deviation), median (95% range) or percentages. Differences in subject characteristics between the groups were evaluated using one-way ANOVA tests for continuous variables and Chi-square tests for proportions.

**Supplementary Table S2. Associations of maternal daily vomiting or nausea during early pregnancy with maternal weight status during pregnancy (N = 4,769)^1^**

| **Maternal vomiting or nausea** | **Prepregnancy BMI (kg/m^2^**) | **Prepregnancy weight (kg)** | **1^st^ trimester weight (kg)** | **2^nd^ trimester weight (kg)** | **3^rd^ trimester weight (kg)** | **Total gestational weight gain (kg)** |
| --- | --- | --- | --- | --- | --- | --- |
| **Daily vomiting** n = 4,769 | 1.44 (1.02,1.87)* | 1.48 (0.16, 2.80) | 0.49 (-0.85,1.84) | -0.02 (-1.27,1.22) | -0.57 (-1.80,0.67) | -1.99 (-2.93,-1.05)* |
| **Daily nausea** n = 4,747 | 0.47 (0.20,0.74)* | 0.56 (-0.21, 1.33) | 0.24 (-0.61,1.08) | -0.14 (-0.93,0.64) | -0.16 (-0.94,0.61) | -0.64 (-1.09,-0.20)* |

^1^Values are regression coefficients (95% Confidence Interval) from univariate linear regression models and reflect the differences in maternal weight status between mothers with and without daily vomiting and nausea in early pregnancy. *P-value <0.05.

**Supplementary Table S3. Associations of maternal daily vomiting or nausea during early pregnancy with maternal nutritional status (N = 4,769) ^1^**

| **Maternal vomiting or nausea** | **Total energy intake (kcal)** | **Total carbohydrates intake (energy %)** | **Total fat intake (energy %)** | **Total protein intake**  **(energy %)** |
| --- | --- | --- | --- | --- |
| **Daily vomiting** n = 4,769 | -120 (-195,-46)* | 1.58 (0.83, 2.32)* | -0.60 (-1.25, 0.05) | -0.81 (-1.10, -0.52)* |
| **Daily nausea** n = 4,747 | -21 (-65,24) | 0.74 (0.30, 1.17)* | -0.38 (-0.76, 0)* | -0.27 (-0.44, -0.10)* |

^1^Values are regression coefficients (95% Confidence Interval) from univariate linear regression models and reflect the differences in multiple parameters of maternal nutritional status between mothers with and without daily vomiting and nausea in early pregnancy. *P-value <0.05.

**Supplementary Table S4. Associations of maternal daily vomiting or nausea during early pregnancy with birth outcomes (N = 4,769)^1^**

| **Maternal vomiting or nausea** | **Birth weight**  **(g)** | **Gestational age at birth**  **(weeks)** | **Gestational age adjusted birth weight (SDS)** |
| --- | --- | --- | --- |
| **Daily vomiting** n = 4,769 | -79 (-131,-27)* | -0.19 (-0.35,-0.02)* | -0.13 (-0.22, 0.03)* |
| **Daily nausea** n = 4,747 | -8 (-41,26) | -0.03 (-0.14,0.07) | 0 (-0.06, 0.06) |

^1^Values are regression coefficients (95% Confidence Interval) from univariate linear regression models and reflect the differences in birth outcomes between women with and without daily vomiting and nausea in early pregnancy. *P-value <0.05. Abbreviations: SDS, standard deviation score.

**Supplementary Table S5. Dose-response associations of maternal vomiting during early pregnancy with childhood general and abdominal fat outcomes (N = 4,760)^1^**

|  | **Childhood fat outcomes**  **Difference in Standard Deviation Score (95% Confidence Interval)** | | | |
| --- | --- | --- | --- | --- |
|  | **Body mass index**  n = 4,760 | **Total body fat mass**  n = 4,627 | **Android/gynoid fat mass ratio**  n = 4,627 | **Abdominal preperitoneal fat mass area**  n = 3,838 |
| **Basic model^2^** |  |  |  |  |
| Daily | 0.26 (0.16,0.35)* | 0.34 (0.25,0.43)* | 0.26 (0.16,0.36)* | 0.24 (0.14,0.34)* |
| Few days a week | 0.08 (-0.01,0.17) | 0.14 (0.06,0.22)* | 0.05 (-0.04,0.14) | 0.15 (0.05,0.24)* |
| Once a week | 0.03 (-0.09,0.14) | -0.01 (-0.12,0.09) | -0.04 (-0.16,0.08) | -0.05 (-0.18,0.07) |
| Less than once a week | -0.03 (-0.12,0.05) | -0.03 (-0.10,0.05) | -0.01 (-0.10,0.07) | -0.07 (-0.16,0.02) |
| Never | *Reference* | *Reference* | *Reference* | *Reference* |
| *Trend^4^* | *0.05 (0.03,0.07)** | *0.07 (0.05,0.08)** | *0.04 (0.02,0.06)** | *0.05 (0.03,0.07)** |
| **Fully adjusted model^3^** |  |  |  |  |
| Daily | 0.08 (-0.01,0.16) | 0.11 (0.02,0.19)* | 0.10 (0,0.20)* | 0.09 (-0.01,0.20) |
| Few days a week | -0.02 (-0.09,0.06) | 0.02 (-0.06,0.09) | -0.03 (-0.12,0.06) | 0.06 (-0.03,0.15) |
| Once a week | -0.04 (-0.14,0.05) | -0.08 (-0.18,0.02) | -0.09 (-0.20,0.03) | -0.09 (-0.21,0.03) |
| Less than once a week | -0.01 (-0.07,0.06) | -0.03 (-0.10,0.04) | -0.01 (-0.09,0.07) | -0.06 (-0.14,0.03) |
| Never | *Reference* | *Reference* | *Reference* | *Reference* |
| *Trend^4^* | *0.01 (-0.01,0.02)* | *0.02 (0,0.03)* | *0.01 (-0.01,0.03)* | *0.02 (0,0.04)* |

^1^Values are regression coefficients (95% Confidence Interval) that reflect the difference for each body fat measure per SDS change between children of mothers within different maternal vomiting categories, as compared to children of mothers who never vomited during early pregnancy. Estimates are based on multiple imputed data. ^2^Basic model is adjusted for child’s sex and age at outcome measurements. ^3^Fully adjusted model includes maternal age, educational level, ethnicity, prepregnancy weight, parity, smoking, folic acid supplement use, diet, delivery mode and pregnancy complications, gestational weight gain, gestational age at birth and birth weight, infant growth: growth in weight from birth until 2 years of age, breastfeeding duration, age at introduction of solid foods and tv-watching. Models for fat mass are additionally adjusted for childhood height. ^4^Tests for trend were based on multiple linear regression models with maternal vomiting during early pregnancy as a continuous variable, from category ‘daily’ through all categories up to category ‘never’. *P-value <0.05.

**Supplementary Table S6. Dose-response associations of maternal vomiting during early pregnancy with childhood cardiovascular risk factors (N = 4,370) ^1^**

|  | **Childhood fat outcomes**  **Difference in Standard Deviation Score (95% Confidence Interval)** | | | |  |
| --- | --- | --- | --- | --- | --- |
|  | **Systolic blood**  **pressure**  n = 4,370 | **Diastolic blood**  **pressure**  n = 4,370 | **Total cholesterol**  n = 3,152 | **Triglycerides**  n = 3,143 | **Insulin**  n = 3,126 |
| **Basic model^2^** |  |  |  |  |  |
| Daily | 0.11 (0.01,0.21)* | 0.12 (0.02,0.22)* | -0.03 (-0.15,0.10) | 0.04 (-0.09,0.16) | 0.09 (-0.04,0.21) |
| Few days a week | 0.01 (-0.09,0.10) | 0.06 (-0.03,0.16) | -0.05 (-0.16,0.06) | -0.08 (-0.20,0.03) | 0.03 (-0.08,0.15) |
| Once a week | 0.12 (-0.01,0.24) | 0.19 (0.07,0.32)* | -0.10 (-0.25,0.05) | 0.06 (-0.09,0.21) | 0.03 (-0.12,0.18) |
| Less than once a week | -0.03 (-0.12,0.06) | 0.03 (-0.06,0.11) | -0.09 (-0.19,0.01) | 0.07 (-0.03,0.17) | 0.07 (-0.04,0.17) |
| Never | *Reference* | *Reference* | *Reference* | *Reference* | *Reference* |
| *Trend^4^* | *0.02 (0,0.04)** | *0.03 (0.01,0.05)** | *-0.01 (-0.04,0.01)* | *0 (-0.03,0.02)* | *0.02 (-0.01,0.04)* |
| **Fully adjusted model^3^** |  |  |  |  |  |
| Daily | 0.02 (-0.09,0.12) | 0.05 (-0.06,0.15) | -0.08 (-0.21,0.05) | 0.01 (0,0.26) | 0.11 (-0.02,0.24) |
| Few days a week | -0.03 (-0.12,0.07) | 0.02 (-0.08,0.12) | -0.07 (-0.18,0.05) | -0.10 (-0.22,0.02) | 0.05 (-0.07,0.16) |
| Once a week | 0.10 (-0.02,0.23) | 0.17 (0.04,0.29)* | -0.12 (-0.27,0.03) | 0.02 (-0.13,0.17) | 0.03 (-0.13,0.18) |
| Less than once a week | -0.03 (-0.12,0.05) | 0.01 (-0.07,0.10) | -0.10 (-0.20,0.01) | 0.07 (-0.04,0.17) | 0.08 (-0.02,0.19) |
| Never | *Reference* | *Reference* | *Reference* | *Reference* | *Reference* |
| *Trend^4^* | *0 (-0.02,0.02)* | *0.01 (-0.01,0.04)* | *-0.02 (-0.05,0)* | *-0.01 (-0.04,0.02)* | *0.02 (-0.01,0.05)* |

^1^Values are regression coefficients (95% Confidence Interval) that reflect the differences in childhood cardiovascular risk factors per SDS change between children of mothers within different maternal vomiting categories, as compared to children of mothers who never vomited during early pregnancy. Estimates are based on multiple imputed data. ^2^Basic model is adjusted for child’s sex and age at outcome measurements. ^3^Fully adjusted model includes maternal age, educational level, ethnicity, prepregnancy weight, parity, smoking, folic acid supplement use, diet, delivery mode and pregnancy complications, gestational weight gain, gestational age at birth and birth weight, infant growth: growth in weight from birth until 2 years of age, breastfeeding duration, age at introduction of solid foods and tv-watching. Models for fat mass are additionally adjusted for childhood height. ^4^Tests for trend were based on multiple linear regression models with maternal vomiting during early pregnancy as a continuous variable, from category ‘daily’ through all categories up to category ‘never’. *P-value <0.05.

**Supplementary Figure S1. An overall conceptual model of the associations between maternal vomiting in early pregnancy with childhood cardiovascular risk factors^1^**

Lifestyle related & socio-demographic factors^2^

*Infant growth & lifestyle^5^*

*Birth outcomes^4^*

*Gestational weight gain^3^*

Maternal vomiting during early pregnancy

Childhood cardiovascular risk factors

*^1^*This figure depicts an overall conceptual model of the associations of maternal daily vomiting in early pregnancy with childhood cardiovascular risk factors with all potential confounders and intermediate covariates for interpretation of the strategy of analysis and results *^2^Lifestyle related and sociodemographic factors* as confounders: child’s sex and age at outcome measurements, childhood height (models for fat mass only), maternal age, maternal educational level, ethnicity, prepregnancy weight, parity, smoking, folic acid supplement use, diet, delivery mode and pregnancy complications. ^3^*Gestational weight gain,* ^4^*birth outcomes:* gestational age at birth and birthweight, and *^5^infant growth and lifestyle:* breastfeeding duration, age at introduction of solid foods, tv-watching and growth in weight from birth until 2 years of age, as intermediate covariates
